# Acidification-induced cellular changes in *Symbiodinium* isolated from *Mussismilia braziliensis*

**DOI:** 10.1371/journal.pone.0220130

**Published:** 2019-08-05

**Authors:** Lilian J. Hill, Wladimir C. Paradas, Maria Julia Willemes, Miria G. Pereira, Paulo S. Salomon, Rodrigo Mariath, Rodrigo L. Moura, Georgia C. Atella, Marcos Farina, Gilberto M. Amado-Filho, Leonardo T. Salgado

**Affiliations:** 1 Diretoria de Pesquisas, Instituto de Pesquisas Jardim Botânico do Rio de Janeiro, Rio de Janeiro, Brazil; 2 Instituto de Biofísica Carlos Chagas Filho, Universidade Federal do Rio de Janeiro (UFRJ), Rio de Janeiro, Brazil; 3 Instituto de Biologia, Universidade Federal do Rio de Janeiro (UFRJ), Rio de Janeiro, Brazil; 4 Instituto de Bioquímica Médica, Universidade Federal do Rio de Janeiro (UFRJ), Rio de Janeiro, Brazil; 5 Instituto de Ciências Biomédicas, Universidade Federal do Rio de Janeiro (UFRJ), Rio de Janeiro, Brazil; Mount Allison University, CANADA

## Abstract

Dinoflagellates from the Symbiodiniaceae family and corals have an ecologically important endosymbiotic relationship. Scleractinian corals cannot survive for long periods without their symbionts. These algae, also known as zooxanthellae, on the other hand, thrives outside the coral cells. The free-living populations of zooxanthellae are essential for the resilience of the coral to environmental stressors such as temperature anomalies and ocean acidification. Yet, little is known about how ocean acidification may affect the free-living zooxanthellae. In this study we aimed to test morphological, physiological and biochemical responses of zooxanthellae from the *Symbiodinium* genus isolated from the coral *Mussismilia braziliensis*, endemic to the Brazilian coast, to acidification led by increased atmospheric CO_2_. We tested whether photosynthetic yield, cell ultrastructure, cell density and lipid profile would change after up to 16 days of exposure to pH 7.5 in an atmospheric *p*CO_2_ of 1633 μatm. Photosynthetic yield and cell density were negatively affected and chloroplasts showed vesiculated thylakoids, indicating morphological damage. Moreover, *Symbiodinium* fatty acid profile drastically changed in acidified condition, showing lower polyunsaturated fatty acids and higher saturated fatty acids contents, when compared to the control, non-acidified condition. These results show that seawater acidification as an only stressor causes significant changes in the physiology, biochemistry and ultrastructure of free-living *Symbiodinium*.

## Introduction

Some marine invertebrates are known to host endosymbiotic dinoflagellates from the family Symbiodiniaceae—also known as zooxanthellae [1–4]. This endosymbiotic relationship is particularly important for reef-building corals because they play a crucial role in the construction of the reef ecosystem, whose maintenance depends on this symbiosis [2,3].

This photosynthetic protist is responsible for providing up to 95% of the energy necessary for the coral, by mediating the translocation of the photosynthetically fixed carbon to glucose, glycerol and amino acids to the host [5,6]. In addition, the symbiont’s cells supply large amounts of oxygen to the host, leading to a significant increase in the rates of mitochondrial respiration, and ATP levels. In scleractinian corals this energy input allows the deposition of calcium carbonate and consequently increases in biomineralization rates [7,8].

These zooxanthellae have two morphologically distinct life stages, one coccoid and one motile [9]. When *in hospite*, they are kept in the cocoid stage and, when free-living, they can be found in either one of these stages. Corals obtain their zooxanthellae either through horizontal or vertical transmissions, depending on the reproduction type: brooders tend to get their symbionts vertically from their progenitors and broadcast spawners tend to acquire the zooxanthellae from the environment [10, 11]. Thus, the availability of free-living populations of zooxanthellae is crucial for both the establishment and the persistence of the symbiosis.

Environmental stressors led by global climate-changing scenarios, such as increased temperature [12] and decreased seawater pH [13], can disrupt the coral-Symbiodiniaceae symbiosis, thus causing the coral bleaching phenomenon [9, 14–16], which can be explained as either the loss of the zooxanthellae by the corals or the degradation of the photosynthetic pigments in the symbiont’s cells [9, 17–19]. Coral colonies can survive bleaching events by reacquiring zooxanthellae [20] and reestablishing their endosymbiosis, but in order to do so, there must be a pool of healthy and viable Symbiodiniaceae cells available in the environment [21].

According to NOAA (National Oceanic and Atmospheric Administration), bleaching events have been reported globally since 1998 due to increased seawater temperature caused by thermal anomalies. The reduction of seawater pH values, caused by increased anthropogenic atmospheric *p*CO_2_ (see prediction scenarios by the Intergovernmental Panel on Climate Change report–IPCC [22]), is also expected to affect negatively coral reefs [12].

In the South Atlantic Ocean (SAO), the Abrolhos Bank off the Brazilian coast represents the largest and richest coralline ecosystem [23, 24] with a high degree of endemism of corals, dominated by one of the oldest and most important genus of scleractinian corals, *Mussismilia* [23, 25]. *Mussismilia braziliensis* is an endemic coral that should soon be listed as an endangered species due to its rapid decline caused by coral diseases and environmental stressors [26, 27]. The Abrolhos Bank has experienced recurrent coral bleaching events and, although many corals were able to recover [28], it is not known whether they would have the same resilience to acidification.

Some studies suggest that the key to resilience in coral colonies lies in their ability to acquire a diverse genotype of Symbiodiniaceae cells, including resistant strains [29–33]. According to recent taxonomic review, the family Symbiodiniaceae now refers to several genera of algae that were previously classified as specific clades of *Symbiodinium sp*.: The genera *Symbiodinium* (formerly Clade A), *Breviolum* (formerly Clade B), *Cladocopium* (formerly Clade C), *Durusdinium* (formerly Clade D) and *Gerakladium* (formerly Clade G) are known for maintaining symbiosis with Cnidarian, like corals and sea anemone [4, 34–37] and are also the most dominant in more severe environments, such as higher irradiance habitats, increased temperature conditions and regions with greater coastal influence [36]

In this context it is known that *M*. *braziliensis* hosts at least two strains of Symbiodiniaceae, corresponding to *Symbiodinium* and *Cladocopium* genera), which could have big implications for the resilience of this species [38].

Many studies concern the effects of environmental stressors in the coral holobiont and, despite knowing that the coral resistance to these stressors depends on the resistance of the zooxanthellae itself, there are few studies related to the responses in a cellular and biochemical level on the symbiont separately. Besides, stressors like acidification could potentially reduce the pool of free-living zooxanthellae on the reef environment for corals to uptake.

In this study we tested the cellular, physiological and biochemical responses of the *Symbiodinium* strain (previously known as clade A4) isolated from *M*. *braziliensis* to seawater acidification caused by increased atmospheric CO_2_.

## Methods

### Isolation and culture establishment

Clonal Symbiodiniaceae cultures were established from populations of *M*. *braziliensis* tissue from collected from Abrolhos Reefs, Brazil as described previously [38]. The strain CCMR0100, which belongs to *Symbiodinium* genus (previously known as clade A4), was obtained from the Culture Collection of Microorganisms at Federal University of Rio de Janeiro (CCMR). Cultures have been kept in sterile f/2 medium [39, 40], prepared with synthetic seawater (Red Sea fish farm LTD., Houston, TX, USA) in a culture chamber with controlled irradiance (photon flux of ca. 80 μmol/m^2^/s, photoperiod of 14-h light/10-h dark) and temperature (24 ± 1°C).

### Experimental design

An acidification assay was performed by exposing *Symbiodinium* cells to a CO_2_ saturated atmosphere for several days. For this study, we tested the worst-case scenario for seawater pH projected for 2100 and beyond [22, 41], 7.5, in comparison to the current seawater pH, 8.1, as a control condition. The target pH for both the control and elevated *p*CO_2_ concentrations were set using the methodology described previously [41], which calculates a seawater *p*CO_2_ of 1638 atm to reach a 7.50 pH and a seawater *pCO*_*2*_ of 464 atm to reach an 8.1 pH. we tested the worst case scenario projected by the IPCC [22] for atmospheric *p*CO_2_ for the year 2100, which corresponds to 1634 μatm *p*CO_2_ and a seawater pH of 7.5, in comparison to current conditions (464 μatm *p*CO_2_ and seawater pH 8.2).

For the experimental design, we used a CO_2_ injection system that measured the atmospheric *p*CO_2_ inside 2 different culture chambers: one corresponding to the control condition and another corresponding to the acidified conditions. This system was projected to inject more CO_2_ whenever the *p*CO_2_ was below the desired. In the control condition, soda lime plates inside filter bags were also used to maintain the lower levels of CO_2_. Then, CO_2_ saturated air inside the culture chambers was pumped into f/2 medium, for 7 days, until the pH levels stabilized in 7.5.

Cells of Symbiodiniaceae strain CCMR0100, corresponding to the genus *Symbiodinium* (previously known as Clade A_4_) were cultured on ten 24-well plates with round coverslips on the bottom of every well for 10 days under normal conditions. Then, cultures were sampled for photosynthetic potential analysis (n = 6), processed for ultrastructure analysis and for cell density (n = 5) and bulked sampled for neutral lipids qualitative (more details and replicate numbers on each specific topic for each analysis). These were considered the before-assay samples (T0).

The T0 cultures were then replicated on ten 24-well plates with the acidified f/2 medium (for the acidified condition) and on ten 24-well plates with non-acidified f/2 medium (for the control condition). Adhered cells were manually and continuously scraped until all visible cell clumps were dissolved. Cultures were then placed in their respective culture chambers (acidified and control) for a total period of 16 days. Samples were collected at 4 different times, depending on the analysis: T1 (after 4 days of incubation), T2 (after 8 days of incubation), T3 (after 12 days of incubation) and T4 (after 16 days of incubation). The photosynthetic potential analysis was done in the before-assay samples and in every one of the times within the assay (T0, T1, T2, T3 and T4), the lipid profile analysis was done on samples collected at T0, T2 and T4 and both ultrastructure and cell density analysis were done before and after the assay (T0 and T4).

To avoid pH elevation caused by photosynthetic CO_2_ uptake, the medium in the cultures were changed every 2 days with the corresponding medium (non-acidified f/2 for control and previously acidified f/2 for the treatment cultures).

Salinity, pH and temperature were measured every two days, before medium replacement, using a multiparameter sensor YSI (YSI, Yellow Springs, OH, USA). The carbonate chemistry of the medium was calculated using CO2SYS [42].

### Photosynthetic potential analysis

The photosynthetic potential was evaluated by pulse-amplitude-modulated (PAM) fluorimetry using an underwater fluorimeter (Diving-PAM, Heinz Walz Gmbh, Effeltrich, Germany) coupled with a light emitting diode (LED, with emission peak at 470nm) and an 8mm fiber optic probe.

Immediately after each collection (at T0, T1, T2, T3 and T4), stationary fluorescence (F) from 6 replicates of each condition and each time point was estimated from the signal obtained under the fluorimeter’s light modulation (pulsed light; intensity < 1μmol/.m^2^/s^1^) and the maximum fluorescence of the light-acclimated sample (Fm") was determined using a pulse of saturating light of short duration (600 ms). The effective quantum yield was calculated by the formula (Fm”—F)/ Fm”. Subsequently, the samples were acclimatized in the dark for 30 minutes, to obtain the maximum potential photosynthetic yield. The intrinsic fluorescence of the dark-adapted sample (Fo) was determined from the signal obtained under the fluorescence modulated light. The maximum fluorescence value (Fm) was measured in the presence of a pulse of saturating light (600 ms, ~ 6000 μmol/m^2^/s^in1^) [43]. Variable fluorescence (Fv) was obtained from the difference between Fm and Fo (Fm-Fo) and the maximum photosynthetic yield calculated by the Fv / Fm ratio.

### Cell density

To analyze cell density differences between the acidified condition and the control at the end of the experiment, coverslips from before and after the experiment were processed for SEM as follow: cells were post-fixed with 2% OsO_4_ (for 1 hour), washed in 0.1M cacodylate buffer and dehydrated in series of ethanol (30, 50, 70, 90 and 3x100%) for 15 minutes each. Then, samples were critical point dried in a Baltec CPD 030, mounted on stubs, sputter-coated with gold and visualized in a Zeiss EVO 40. Ten images of random parts of five different stubs for each condition were taken at the same magnitude (800X). Each stub corresponded to one replicate and each image to a sub-replicate. The images were used to count the number of cells on each coverslip. Cell counting was made using SEM images of same magnitude. Considering that the area of each field analyzed was 80000 μm^2^, total cell densities of each condition were calculated by multiplying the number of cells per μm^2^ by the area of the coverslips. Cell densities were then calculated for each sample of each treatment and for the before-assay samples (T0). The intrinsic rate of increase of both conditions was further calculated according to Gotelli (1995) [44].

### Structural analyses

In order to evaluate structural and ultrastructural changes on *Symbiodinium* cells due to acidification treatment, we subjected the samples from T0 and T4 incubated in both acidified and non-acidified conditions to laser scanning confocal microscopy (LSCM) to access changes on lipid storage and chlorophyll fluorescence, and to transmission electron microscopy (TEM) to determine possible organelle changes.

#### Transmission electron microscopy

The samples from T0 and T4 of both conditions were fixed as described by previous study [45].Here we added the volume of 6 wells (18ml), per replica, to 18 ml of a fixation solution (0.1M sodium cacodylate buffer containing 2% glutaraldehyde and 0.3 M sucrose). In total, we used four replica for each treatment (before assay, control and acidified). Samples were fixed for 1 hour at room temperature, and then washed in four changes of 0.1 M sodium cacodylate buffer with decreasing sucrose concentration: 0.3 M, 0.15 M, 0.075 M (up to 0 M), 20 min in each step. Next, samples were post-fixed with 2% OsO_4_ with water for 1 hour; dehydration was then performed in acetone series (30%, 40%, 50%, 70%, 80%, 90% and 3x100% - 20 minutes each) and samples were embedded in Spurr resin. Ultrathin sections (~70 nm) were obtained on a Leica EM UC7 ultramicrotome with a diamond knife (Diatome, Hatfield, PA, USA) and collected on 300 mesh copper grids. After staining in uranyl acetate and lead citrate, sections were examined in the EM 900 Zeiss.

#### Confocal laser scanning microscopy

For LSCM analysis, cells adhered on coverslips from T0 and T4 of both conditions were fixed with 4% formaldehyde in seawater for 1 hour and incubated with 10 μg/ml Nile Red (Sigma-Aldrich, St. Louis, Missouri, USA) for 30 minutes, then washed twice in PBS (pH7.4). The coverslips were then mounted on slides with Fluoroshield^TM^ with DAPI (Sigma-Aldrich) and taken to a TCS SPE microscope (Leica Microsystems, Wetzlar, Germany). The excitation wavelengths were 405 nm and 488 nm and fluorescence emission peaks were: 440 nm (DAPI), 535 nm (Nile Red) and 650 nm (Chlorophyll a). Images acquisition resulting resolution was 2,048 x 2,048 and, to improve the image quality, they were processed by 3D deconvolution with LAS AF software (Leica Microsystems Company).

### Lipid analysis

To access the effect of acidification on the lipid content produced by *Symbiodinium* cells, we performed a qualitative lipid analysis on cells from both control and acidified conditions (T0, T2 and T4), as followed:

*Neutral lipid extraction*—Lipids from *Symbiodinium* samples (T0, T2, and T4 from all conditions) were extracted according to previous studies [46]. Then, samples were submitted to gas chromatography with mass spectrometry (GC/MS) for fatty acid and sterol composition analysis.

*Fatty acid composition*–For FA analysis, the neutral lipid extracts were subjected to the same protocol used previously (see [47]) and modified by (see [48]). The analysis was performed with a Shimadzu QP2010 Plus instrument equipped with a mass spectrometry detector (Shimadzu Corporation, Kyoto, KR, Japan) and a Hewlett-Packard Ultra 2 polysiloxane capillary column (Hewlett-Packard Company, Palo Alto, CA, USA) (25 m x 0.20 mm i.d. x 0.33 μm). The injector was maintained at 250°C with a gas split flow rate of 1:1. The column oven temperature was programmed to increase from 40°C to 160°C (rate: 30°C min^-1^); from 160°C to 233°C (rate: 1°C min^-1^) and from 233°C to 300°C (rate: 30°C min^-1^), and, at the end, the temperature was maintained at 300°C for 10 min. Helium was used as a carrier gas at a flow rate of 20.5 mL.min^-1^. Electron impact spectra were recorded at 70 eV with a scan time of 1 s. The FA species were identified by comparing their mass spectra with the mass spectra of FAME 37-methylated FA mix standards (Supelco, Sigma-Aldrich Company, Saint Louis, MO, USA). GC-MS solution software version 2.53 (Shimadzu Corporation, Kyoto, KR, Japan) was used for data processing.

*Sterol Composition*—The sterol content was evaluated by Gas chromatography–mass spectrometry analysis (GC/MS). Extracts were submitted prior to saponification according to Arthington-Skaggs and co-workers [49], and then to GC-MS as described in previous study by our group [48]: The GC-MS was equipped with a polysiloxane capillary column RTX-1 MS (30 m x 0.25 mm i.d. x film thickness 0.25 μm) (Restek Corporation, Bellefonte, PA, USA). The injector was maintained at 250°C with a gas split flow rate of 1:1. The column oven temperature was programmed to increase from 120°C to 250°C (rate; 20°C min^-1^), from 250°C to 280°C (rate; 5°C min^-1^) and from 280°C to 300°C (rate; 10°C min^-1^), and, at the end, the temperature was maintained at 300°C for 6 min. Helium was used as a carrier gas at a flow rate of 15.6 mL.min^-1^. Electron impact spectra were recorded at 70 eV with a scan time of 1 s. The sterol species were identified by comparing their mass spectra with the mass spectra of a specific standard mix (Supelco, Sigma-Aldrich). In addition, for some specific *Symbiodinium* sterols identification, the retention times of the present data and from literature (CS-155; [50]) were used to identify sterol species.

#### Lipid droplets saturation

Images of TEM were used to comparatively analyze the saturation levels on lipids inside lipid droplets. Osmium tetroxide (OsO_4)_ is widely used to fix lipid molecules, and it is known to react with unsaturated lipids [51]. Compartments with high electron density usually present more unsaturated fatty acids, whereas electron lucent subcellular structures are related to low levels of unsaturated lipids. All TEM images were obtained using similar acquisition conditions, as e.g. time exposure, camera sensitivity, image gamma correction. Sections thickness of all samples analyzed was ~60nm and, to reduce the effect of section thickness variations on image contrast, each image was obtained from a different section. To the normalization of the gray values measurement, resin section regions near cells, without any biological material visible, where used as white reference, *i*.*e*. corresponding to gray values near zero. Eighteen LD’s from five different cells from each condition were analyzed. Therefore, lipid droplets gray values were measured using Image J [52, 53] to compare the degree of unsaturation inside lipid droplets from *Symbiodinium* cells under acidified and control conditions. We analyzed all the lipid droplets observed in 5 cells in each one of the four replicates (n = 4).

### Statistical analysis

Results for the photosynthetic potential were submitted to permutational analysis of variance (PERMANOVA) to check if the factors pH and time, interacted and post hoc t-tests used to evaluate differences in conditions and time. These analyses were done using the software PRIMER-E.

Cell density and lipid droplets saturation level data were submitted to one-way ANOVA followed by Tukey’s multiple comparisons test using GraphPad Prism version 8.0.0 for windows (GraphPad Software, San Diego, California USA). Since the fatty acids and sterol profiles show qualitative results, they were submitted to independent Bray-Curtis Similarity analysis (cluster) using the software PRIMER-E.

## Results

### Acidification affects carbonate chemistry in seawater

Salinity, temperature and total alkalinity did not vary significantly between the control and acidified conditions along the experiment. However, the atmospheric *p*CO_2_ influenced not only the water pCO_2_, but also the HCO_3_ and the CO_3_ concentrations, as well as the aragonite and calcite saturation states (Table 1).

**Table 1:** Carbonate chemistry from control and acidified conditions. Average levels of *p*CO_2_ from air and water, pH, HCO_3_, CO_3_, aragonite saturation state (Ω_arag_), calcite saturation state (Ω_calc_), total alkalinity (TA), temperature and salinity from control and acidified conditions throughout the experiment.

	Control	Acidified
***p*CO_2_ air (μatm)**	483.87 ±0.90	1633.25 ±1.00
***p*CO_2_ water (μatm)**	244.13 ±34.78	1403.81 ±111.11
**CO**_**2**_ **water (μmol kg** ^**-1**^**)**	7.18 ±1.05	40.83 ±3.16
**pH** **(total hydrogen scale)**	8.24 ±0.04	7.59 ±0.03
**HCO**_**3**_^**-**^ **(μmol kg** ^**-1**^**)**	1718.40 ±100.61	2210.10 ±44.30
**CO**_**3**_^**-**^ **(μmol kg** ^**-1**^**)**	313.70 ±16.41	92.70 ±9.01
**Ω**_**arag**_	4.86 ±0.27	1.43 ±0.12
**Ω**_**calc**_	7.41 ±0.41	2.17 ±0.19
**TA** **(μmol kg** ^**-1**^**)**	2487.5 ±65.62	2437.5 ±46.87
**Temperature (**^**o**^**C)**	23.03 ±0.44	23.34 ±0.48
**Salinity (PSU)**	36.87 ±1.9	37.50 ±2.12

### Photosynthetic potential is affected by changes in pH according to time

**Fig 1:**
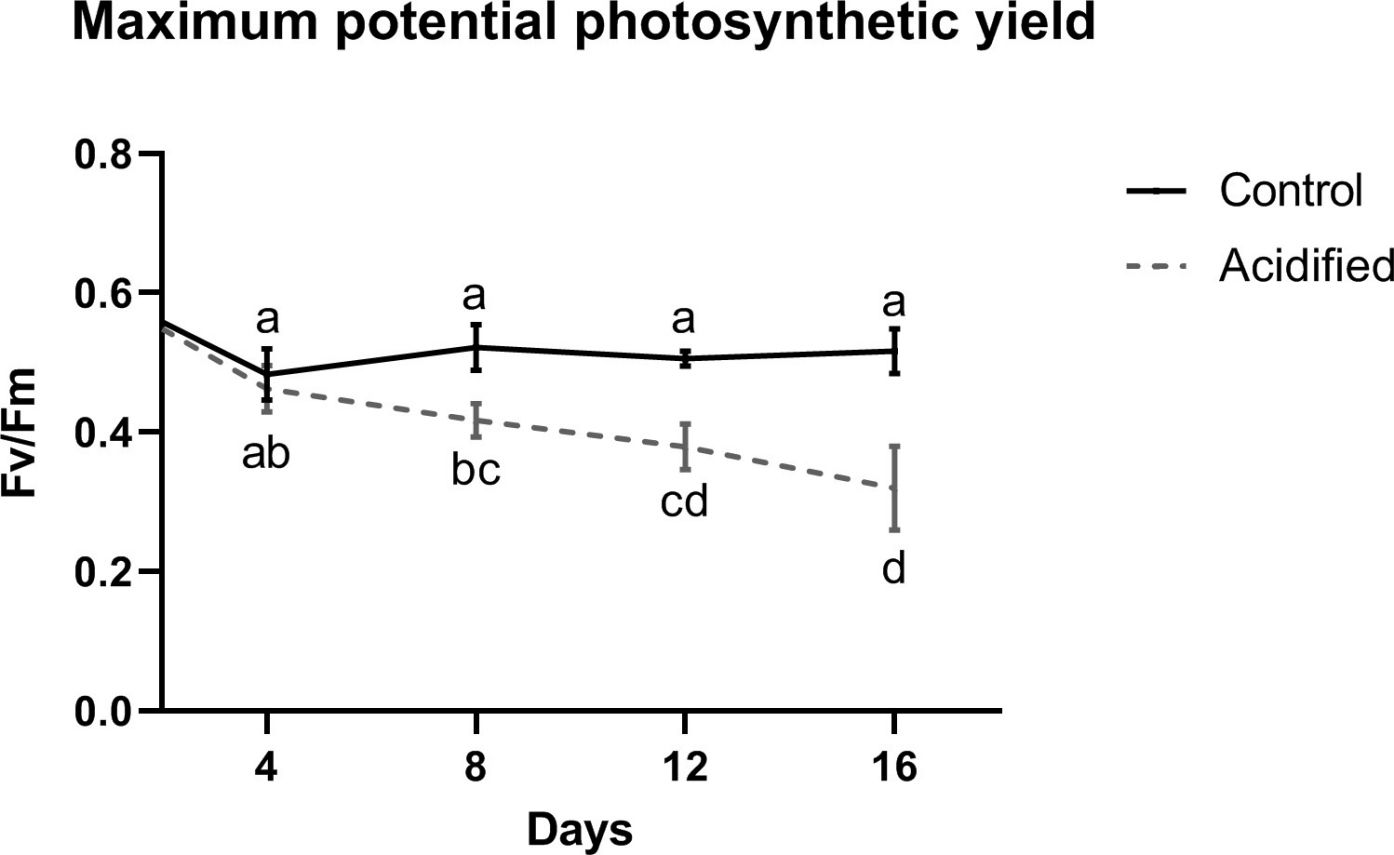
Maximum potential photosynthetic yield of *Symbiodinium* cells. Measurements were made on control and acidified samples after 4, 8, 12 and 16 days of incubation. Photosynthetic yield starts dropping henceforth 8 days of incubation and continues to diminish until the T3 (12 days), comparing to the control conditions. Besides, photosynthetic yield on control samples remains the same throughout the whole experiment. (n = 6).

Samples acclimatized in the acidified condition had a decrease on their maximum potential photosynthetic yield (Fv/Fm) starting from 8 days (T2) until 12 days (T3) of incubation (Fig 1). The 16 days, i.e., T4 samples from the acidified condition had no significant difference when compared with T3. Samples from control condition showed no significant differences along all the experiment time. Moreover, the PERMANOVA test pointed to a significant interaction between pH and time (p<0.001) (S1 Table). Data for the effective quantum yield (ΔF/Fm’) showed similar pattern, with a decrease from 8 days of acidification (p<0.0001; S1 Fig).

### Acidification influences the *Symbiodinium* cell proliferation

**Fig 2:**
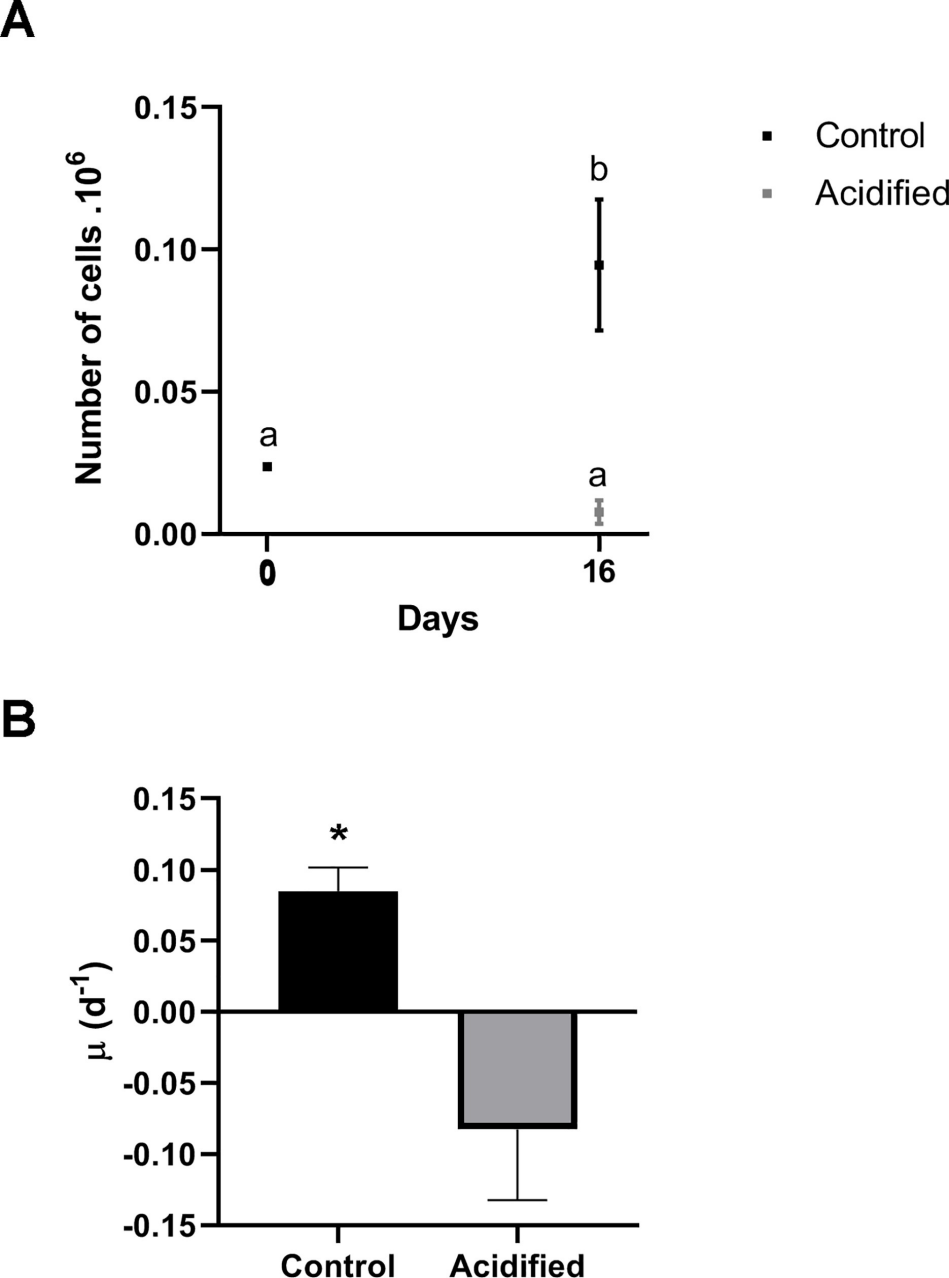
*Symbiodinium* cell proliferation according to pH environment. A–*Symbiodinium* density: Samples incubated in the acidified condition after 16 days showed no significant difference in comparison to T0 (grey squares) (p = 0.1875). On the other hand, cells from control (black squares) had an increase of cell density after 16 days of incubation (p< 0.0001). Different letters above the points show significant difference. B–Intrinsic Rate of Increase of *Symbiodinium* populations submitted to control and acidified conditions after 16 days. The asterisk represents significant difference (p = 0.0011) (n = 5).

At the beginning of the incubation (T0), total cell density was around 2.4 x 10^4^ cells for each condition. At the end of the assay, the acidified condition presented a cell density of 0.8 x 10^4^ cells, representing a decrease of 66.6%. On the other hand, in control assays, it was found 9.0 x 10^4^ cells, and the input of cell population was estimated in 3.75 fold (Fig 2A). Although our statistical analysis did not point significant difference between T0 and acidified samples, our calculations showed that the intrinsic rate of increase where significantly different between conditions: control samples had ~ 0.084 rate, and the acidified samples had ~ -0.082 rate (Fig 2B).

**Fig 3:**
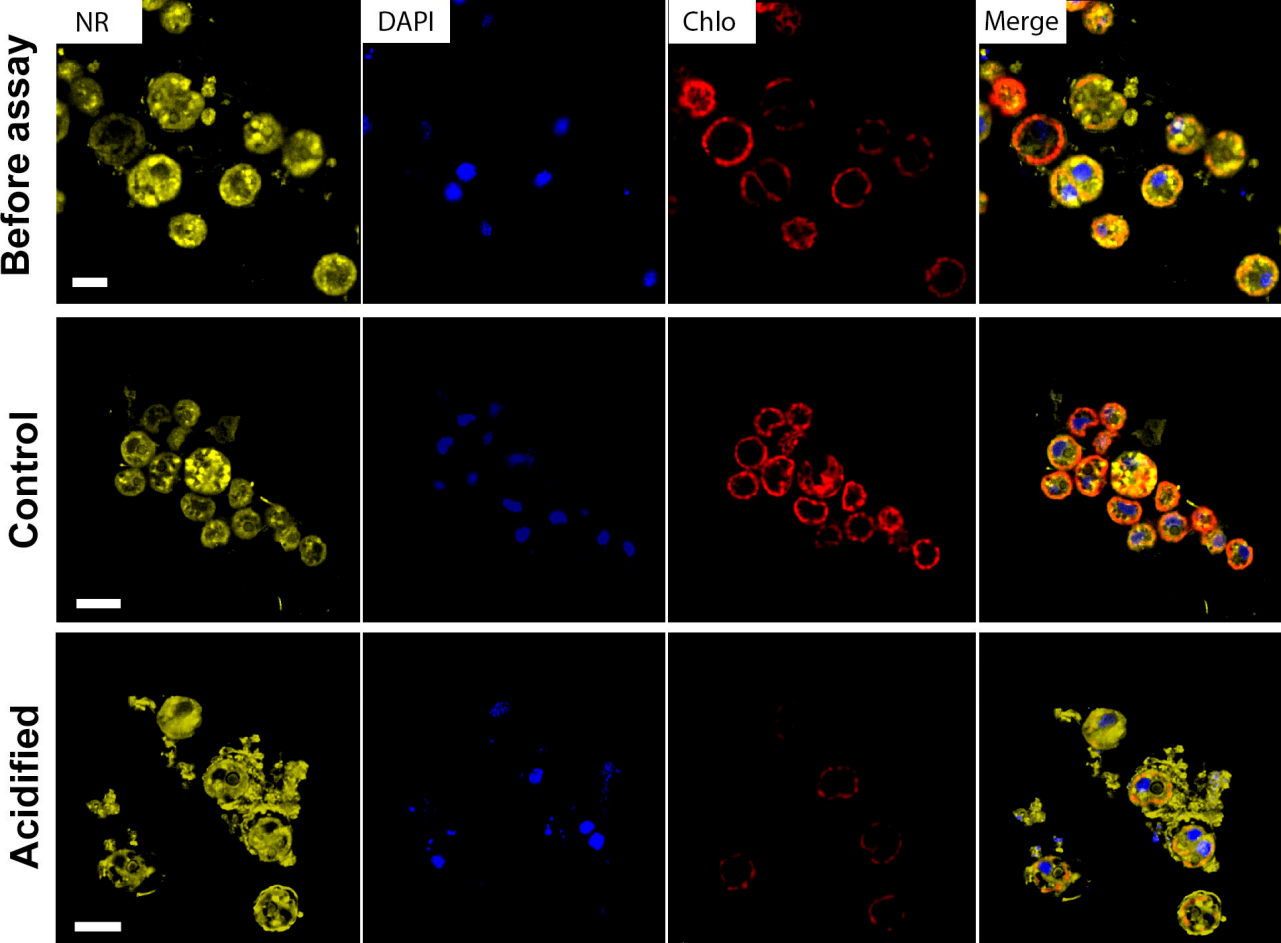
Confocal laser scanning micrographs. A—Cells before the acidification assay showed typical intensity of chlorophyll autofluorescence and distribution of neutral lipids (marked with Nile Red in yellow). B—Cells from control samples after 16 days of incubation showed similar pattern of chlorophyll autofluorescence (red) and neutral lipids intensity and distribution, comparing to the T0 samples. C—Cells from acidified samples after 16 days showed low-intensity autofluorescence from the chlorophyll and diffuse and extracellular Nile Red labeling. Red = chlorophyll; Blue = nuclei marked with DAPI; Yellow = Neutral lipids marked with Nile Red. Bars = 5μm.

Moreover, in the CLSM images we could observe the localization of neutral lipids stocks by NR (Nile Red) staining. There was a clear difference between both before the assay and control comparing to acidified samples (Fig 3). Control and cells before the assay showed intense chlorophyll fluorescence and its regular distribution, in addition to individualized intracellular NR fluorescence. On the other hand, acidified cells presented weak chlorophyll fluorescence and a diffuse, extracellular and less individualized NR staining, which can indicate a beginning of cell death process.

### Acidification of seawater causes ultrastructural damages in *Symbiodinium* cells

**Fig 4:**
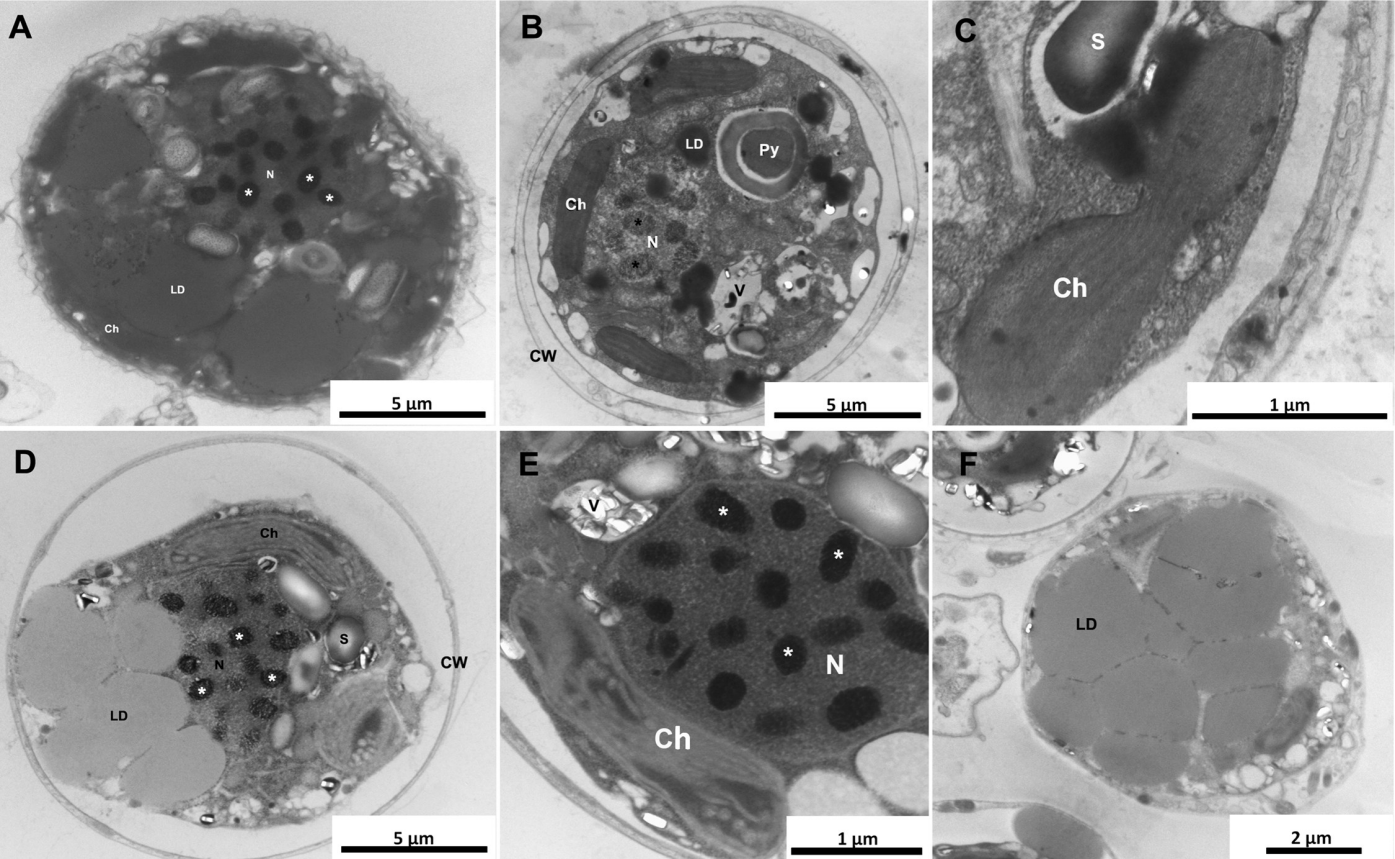
Ultrastructure of *Symbiodinium* cells under different pHs. A–Cells before the acidification assay presented typical organelles, such as nucleus (with condensed chromosomes), electron dense lipid droplets and reticulated chloroplast as normally described in *Symbiodinium* cells. B and C—control condition samples had cells similar to those before the beginning of experiment with typical nucleus, chloroplast, lipid droplets, pyrenoid and vesicles with uric acid deposits from the eyespot (B). C–Higher magnification to show an intact chloroplast. D—F–Ultrathin sections of *Symbiodinium* cells after acidification. Alterations in electron density of lipid droplets indicate a lower content of unsaturated fatty acids (D, F) and damaged chloroplasts with disorganized thylakoid membranes were also observed (D and E). N = nucleus; Ch = chloroplast; LD = lipid droplets; Py = pyrenoid; V = vesicles; S = starch grain; CW = cell wall; Asterisk = condensed chromosomes.

The analysis of *Symbiodinium* cells by electron microscope revealed damaged chloroplasts with vesiculated thylakoids on acidification samples, after 16 days of seawater acidification (Fig 4D–4F). Cell membrane was frequently observed detached from cell wall. Moreover, we found cells highly vesiculated indicating cell death and lipid droplets with lower electron density when compared to control. Other structures such as nucleus, condensed chromosomes and pyrenoid did not suffer any significant structural changes. Control cells and cells from before the assay showed regular shaped organelles (Fig 4A–4C), such chloroplast with organized thylakoid membranes (Fig 4C) and darker lipid droplets (indicating more unsaturated lipids in the core).

### Acidification of seawater alters the fatty acid saturation in *Symbiodinium* cells

**Fig 5:**
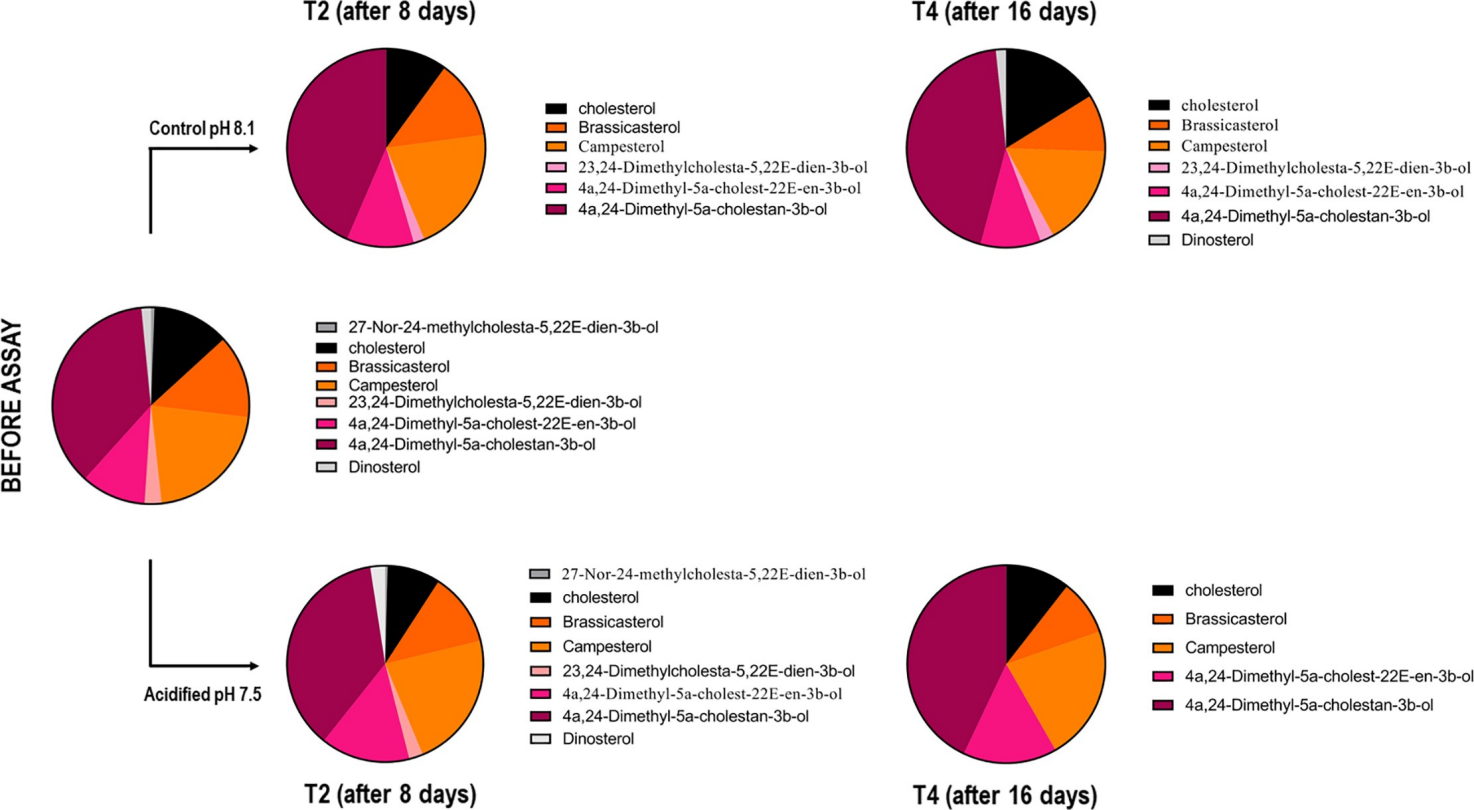
Sterol qualitative profile in *Symbiodinium* cells submitted or not to seawater acidification. Sterol species detected in cells during 0, 8 and 16 days in control (pH 8.1) or acidified (pH 7.5) conditions. No major differences were seen between T0, control and acidified conditions.

**Fig 6:**
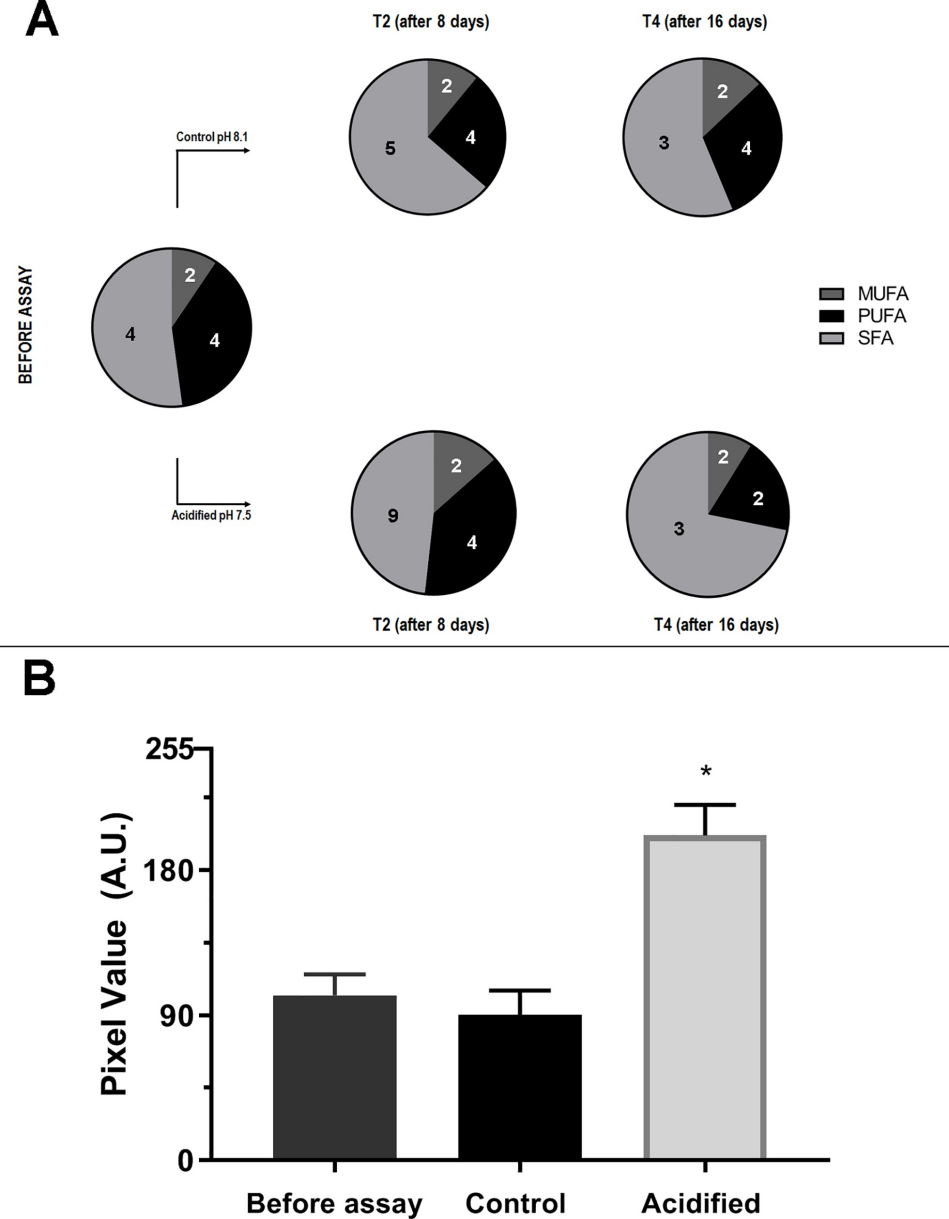
Proportion of different FAs classes in *Symbiodinium* cells before the assay and control and acidified conditions after 8 (T2) and 16 (T4) days. (A) Distribution of MUFAs, PUFAs and SFAs in cells before assay and during 8 and 16 days of control or acidified conditions. Numbers of chemical FA species of each class are presented within pie charts. The increase of SFA in acidified seawater is correlated to increase of electron lucent LDs. (B). Lipid Bodies saturation levels in samples from before the assay and 16 days after acidified and control conditions. Pixel values refers to brightness in LD’s, 255 corresponds to white and 0 corresponds to black. Cells cultivated in acidified seawater present more saturated LDs than ones kept in control pH. Asterisk represents significant difference (p<0.001; n = 4).

**Fig 7:**
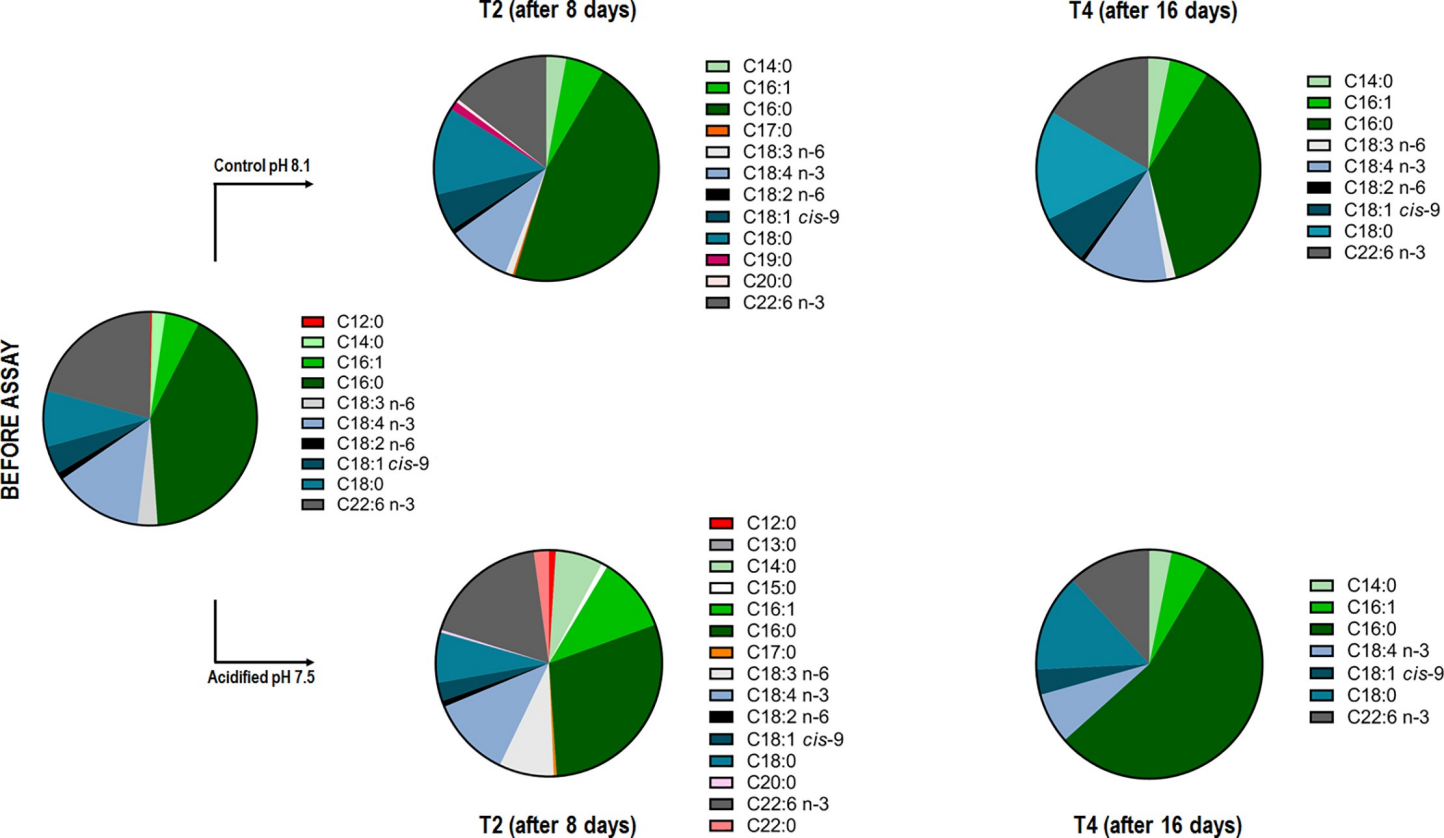
Fatty acid qualitative profile in *Symbiodinium* cells submitted or not to seawater acidification. Fatty acid species detected in cells during 0, 8 and 16 days in control (pH 8.1) or acidified (pH 7.5) conditions. The decrease of species number was evident in the acidified condition after 16 days (T4), in comparison to T0 and T4 of control condition.

We performed GC-MS analysis in order to determine the qualitative profile of fatty acids and sterols of *Symbiodinium* cells under control and acidified conditions before the assay and after 8 and 16 days of assay (Figs 5–7). In total we identified 8 different sterol species (Fig 5) and 16 different fatty acids (FA) species, which of 10 saturated (SFA), two monounsaturated (MUFA) and four polyunsaturated (PUFA) (Figs 6 and 7).

Regarding the sterol analysis, on the before the assay (T0) samples, the *Symbiodinium* cells presented 8 sterol species, with 4a,24-dimethyl-5a-cholestan-3β-ol and 24-methylcholest-5-en-3β-ol (campesterol) as the more abundant sterols (36.4% and 21.23%, respectively) (Fig 5). After 8 days, acidified samples presented similar sterol profile regarding both species number and proportion. Samples after 16 days of acidification did not show three of the eight sterol species that were present in the T0 samples, including Dinosterol. Although cells from control condition after 8 days also did not present Dinosterol, samples from the control condition after 16 days showed this sterol species at a similar proportion as samples from the T0. Bray-Curtis analysis showed no similarity pattern between conditions (S1 Fig).

In relation to the fatty acid (FA) analysis, we found a difference in PUFA (polyunsaturated fatty acids) number amongst the conditions: before-assay samples (T0) and control samples after 8 and 16 days (T2 and T4) showed four PUFA species (C18:3 n-6, C22:6 n-3, C18:2 n-6 and C18:4 n-3), and acidified samples after 16 days (T4) showed only two PUFA species (C18:4 n-3 and C22:6 n-3) (Fig 6A). In addition, acidified cells after 8 days showed more species of FA, with an increase of SFA species (Fig 6A). After 16 days, samples from the acidified condition showed a decrease of both PUFA and SFA species; however the percentage of SFA seemed higher in comparison to control conditions (T0, T2 and T4).

Moreover, the lipid droplets’ (LD) analysis showed that after 16 days, cells from acidified condition had more electron-lucent LD’s (Fig 6B), indicating a more saturated content and corroborating with the FA qualitative profile.

The FA profile analysis revealed that, in addition to increasing the saturation levels of the FA, the acidified condition also decreased the number of total FA species from 10 species on the T0 samples to 7 species on the T4 samples (Fig 7). However, on the T2 samples, we found not only the highest FA diversity (with 15 species), but also FA species that we did not find in any other samples: C13:0, C15:0 and C22:0 (Fig 7).

Despite of the decrease of FA diversity in the acidified samples after 16 days, there was an apparent percentage increase in saturated FA, such as C14:0 (57.1%), C16:0 (32.7%) and C18:0 (11.82%), which could be responsible for the more electron-lucent lipid droplets seen on the LD’s saturation analysis (Fig 6). Interestingly, C18:1 cis-9 increased 70.42% in control and reduced 16% in acidified condition. The long-chain fatty acid C20:4 and C18:3 were absent in the samples from the acidified condition after 16 days. C22:6 reduced 20.6% in control and 42.6% in acidified condition. Although some SFA species only occurred in the samples from the acidified condition after 8 days incubation (C13:0, C15:0 and C22:0), they had little contribution to the total FA composition with 0.09%, 0.85% and 2.16% respectively.

Moreover, Bray-Curtis analysis showed a high similarity (>85%) between samples from the T0 and from the control conditions (T2 and T4). While samples from the acidified condition were grouped apart from control samples (S2 Fig).

These results led us to assume that the fatty acid metabolism is affected by changes in pH environment.

## Discussion

In this study we could reproduce the seawater acidification by the increase of atmospheric CO_2_ leading to several changes in ultrastructure, biochemical and physiological conditions of free-living *Symbiodinium* cells. The assay performed here mimicked the most drastic scenario predicted by the IPCC for atmospheric *p*CO_2_ for the year 2100, by constant injection of atmospheric CO_2_, which dissolved into the water decreasing its pH similarly to what is expected to happen *in situ*.

### Acidification reduces photosynthetic efficiency and growth of *Symbiodinium*

Photosynthetic potential of free living *Symbiodinium* decreased drastically when organisms were submitted to the acidified condition, indicating physiological damage of the photosynthetic machinery. This result combined with the TEM images showing damaged chloroplasts indicates that seawater acidification causes cellular damage in a level that can affect essential physiological functions. Although it is well known that high temperatures cause physiological changes in different genera of Symbiodiniaceae [54, 55], our study showed that low pH due to high *p*CO_2_ alone also causes changes on the physiology of free-living *Symbiodinium*.

It is known that dinoflagellates like *Symbiodinium* have the RuBisCO enzyme type II, which is less efficient to fix carbon than other types of the same enzyme [56]. Therefore, these cells have carbon concentration mechanisms (CCM) which are essential for a high photosynthetic yield [57–58]. A previous study [59] has shown that free-living *Symbiodinium* cells (formerly known as clade A2) had a higher photosynthetic yield on pH 7.7, suggesting that a higher *p*CO_2_ could be beneficial to *Symbiodinium* cells. However, our study showed that after 16 days at pH 7.5 there are enough structural damage on the chloroplast to make the photosynthetic yield to decrease drastically on *Symbiodinium*. This could be due to less RuBisCO available or even to a less efficient CCM on this particular strain. Concerning the response of *in hospite* Symbiodiniaceae to an acidic condition, It has been previously demonstrated that the low pH of symbiosome lumen can be beneficial as it promotes the conversion of HCO_3_^-^ to CO_2_, which occurs almost instantaneously in the presence of carbonic anhydrases [60]. This process increases the local concentration of CO_2_ surrounding the algal cell (in symbiosome lumen), which then diffuses across the cell wall and plasma membrane of the symbiont. Therefore, a low pH condition may not be the unique stressing factor to free-living zooxanthellae. Maybe, the negative response to acidification treatment can be also attributed to other changes in seawater chemistry, to the uncontrolled increase of dissolved CO_2_ and to the absence of cellular modifications like those allowing the survival inside host cells and induced by the symbiotic process.

A recent study showed that treatment with high *p*CO_2_ after 12 days reduced 35% of the division rates of symbionts in *Seriatopora caliendrum* corals [61]. Here we found a clear growth inhibition on free living *Symbiodinium* submitted to acidified condition. Moreover, our data showed a negative intrinsic rate of increase on the acidified condition, indicating a population decline. Similarly, previous study showed that the cell cycle dynamics of cultured Symbiodiniaceae also was influenced by heat stress, which specifically caused the cell cycle arrest in G_1_ phase [62]. Apart from that, membrane debris observed on CMLS images with NR staining and highly vesiculated cells seen on TEM images also indicate cell death. These results suggest that longstanding exposure to high *p*CO_2_ and low pH inhibits cell proliferation and, possibly, induces cell death processes on *Symbiodinium*.

### Changes in chloroplast ultrastructure and lipid content and profile

The chloroplast is known to play an important role on FA biosynthesis in microalgae, where the elongation and desaturation of the carbon chain of fatty acids occurs [63, 64]. Here we found that, after 16 days, the samples from the acidified condition had a decrease of PUFA diversity and content. This, combined with the damaged chloroplasts seen on TEM images, suggests that high *p*CO_2_ atmospheric level affected the chloroplasts to the point of impair the normal FA synthesis. Moreover, some PUFA play important roles in plant cells by protecting and helping the fluidity of the thylakoids membranes and the electron flow between electron acceptors of photosystem II [65–67]. This could explain the apparent increased percentage of PUFA in the acidified samples from 8 days as an initial response of the cell that might have resulted in the protection of the chloroplast from the cell stress caused by the acidification. Thus, we show that, at least in this particular strain, free-living Symbiodiniaceae could resist a lower pH in a short-term period. Furthermore, the LD’s saturation analysis showed a higher saturated content on acidified samples after 16 days, which corroborates to the decrease in PUFA percentage and diversity and the increase in SFA.

The n-6 FA C18:2n-6 and C18:3n-6 are known to be important precursors of the long-chained FA C22:4n-6 (Arachdonic acid), which is responsible for water transport across membranes and an important component of the immune response in the host’s cells [68, 69]. Moreover, these n-6 FA, as well as n-3 FA (such as C18:4n-3 and C22:6n-3) are known to be translocated from symbionts to hosts [70, 71]. Therefore, it has been recently suggested that an appropriate balance of n-3 and n-6 is crucial for the coral holobiont health [68, 69] and, hence, the n-3:n-6 ratio could be a good putative FA indicator [72]. Our data showed that both of the n-6 PUFA that were present in all samples disappeared after 16 days on the acidified condition. This indicates that acidification might interfere not only in the synthesis of essential FA, but also in the free-living symbiont’s health as a hole. Moreover, these results, combined with the lipid droplets saturation analysis suggest that, at lower PH, Symbiodiniaceae from the genus *Symbiodinium* reduce their capacity of producing unsaturated FA.

A previous study [73] has shown that high temperature enhances photodamage of the photosynthetic machinery of *Symbiodinium* and inhibits its repair, which requires *de novo* PUFA synthesis [74]. We hypothesize that this also happens with *Symbiodinium* cells under low pH, i.e. the ability of *Symbiodinium* to synthesize new PUFA is reduced due to damage in the thylakoid membranes. Therefore, the photosynthetic machinery cannot be repaired leading photosynthetic yield to drop.

Moreover, it has been shown that lipid content varies with the cell cycle of the dinoflagellate *Crypthecodinium cohnii* [75] while another study has shown that FA inhibition causes G1 arrest or a transition delay from S to G2/M and G2/M to G1 on Symbiodiniaceae [76]. These works are in agreement with the G_1_ arrest caused by heat stress observed in cultured *Symbiodinium* [62]. Considering our results that revealed severe photosynthesis impairment and a decrease of important FA species, the inhibition of population growth on the acidified condition could be related to cell cycle arrest. On the other hand, a broad programmed cell death event cannot be disconsidered once diverse environmental stress can lead to this cell condition [77] and also because of the altered chloroplast morphology, considered a hint of this process [78], in *Symbiodinium* cells submitted to water acidification. Finally, although Bray-Curtis analysis showed no similarity patterns amongst the samples regarding sterol composition, Dinosterol was not found in samples from acidified condition after 16 days. Dinosterol is considered a dinoflagellate biomarker [79] and the inability to produce it by cells submitted to low pH for a longer period of time may suggest a change in sterol metabolism.

## Conclusion

The present study gave first evidence of how free-living zooxanthellae may respond to future ocean acidification due to increase of atmospheric CO_2_. Our results showed that high *p*CO_2_ atmospheric level and low pH levels alone can cause significant changes in the physiology, biochemistry and ultrastructure of free-living Symbiodiniaceae from the *Symbiodinium* genus. Thus, potentially affecting free-living populations of these symbionts, which are crucial for coral reefs resilience.

Despite the new insights our results have brought, further studies are necessary to fully understand biochemical changes in Symbiodiniaceae submitted to the conditions climate change may lead to.

## Supporting information

S1 Fig Effective quantum yield of *Symbiodinium* cells. Measurements were made on control and acidified samples after 4, 8, 12 and 16 days of incubation. Effective quantum yield starts dropping henceforth 8 days of incubation and continues to diminish until the T4 (16 days), comparing to the control conditions (n = 6; p<0.001).
(TIF)

S2 Fig Similarity of sterol composition. T0 = before assay samples, T2 = 8 days after incubation, T4 = 16 days after incubation. There was no similarity pattern amongst the samples.
(TIF)

S3 Fig Similarity of fatty acid composition. T0 = before assay samples, T2 = 8 days after incubation, T4 = 16 days after incubation.
(TIF)

S1 Table Photosynthetic yield. Statistical results of PERMANOVA.
(DOCX)

S2 Table Cell density. Statistical results of One-way Anova.
(DOCX)

S3 Table Statistical results of Tukey HSD post-hoc for cell density. A = before assay samples; B = control samples; C = acidified samples.
(DOCX)

S4 Table Lipid droplets saturation statistics. Results of One-way Anova for saturation inside lipid droplets.
(DOCX)

S5 Table Post hoc Tukey HSD results for saturation inside lipid droplets. A = before assay samples; B = control samples; C = acidified samples.
(DOCX)

S1 Dataset Raw data used for all analysis. Each sheet corresponds to raw data used for each analysis: carbonate chemistry (Carbonate Chemistry), maximum potential photosynthetic yield (Photosynthesis_FvFm), effective quantum yield (Photosynthesis_Yield), cell density (Cell Density), sterol composition (Lipid analysis_Sterol), fatty acids composition (Lipid analysis_FA) and lipid bodies saturation levels (Lipid analysis_LD saturation).
(XLSX)
