# Supplementary figures and images for: Acidification-induced cellular changes in Symbiodinium isolated from Mussismilia braziliensis
Source: PLoS One. 2019 Aug 5;14(8):e0220130. doi: 10.1371/journal.pone.0220130 (PMC6681953; doi:10.1371/journal.pone.0220130)

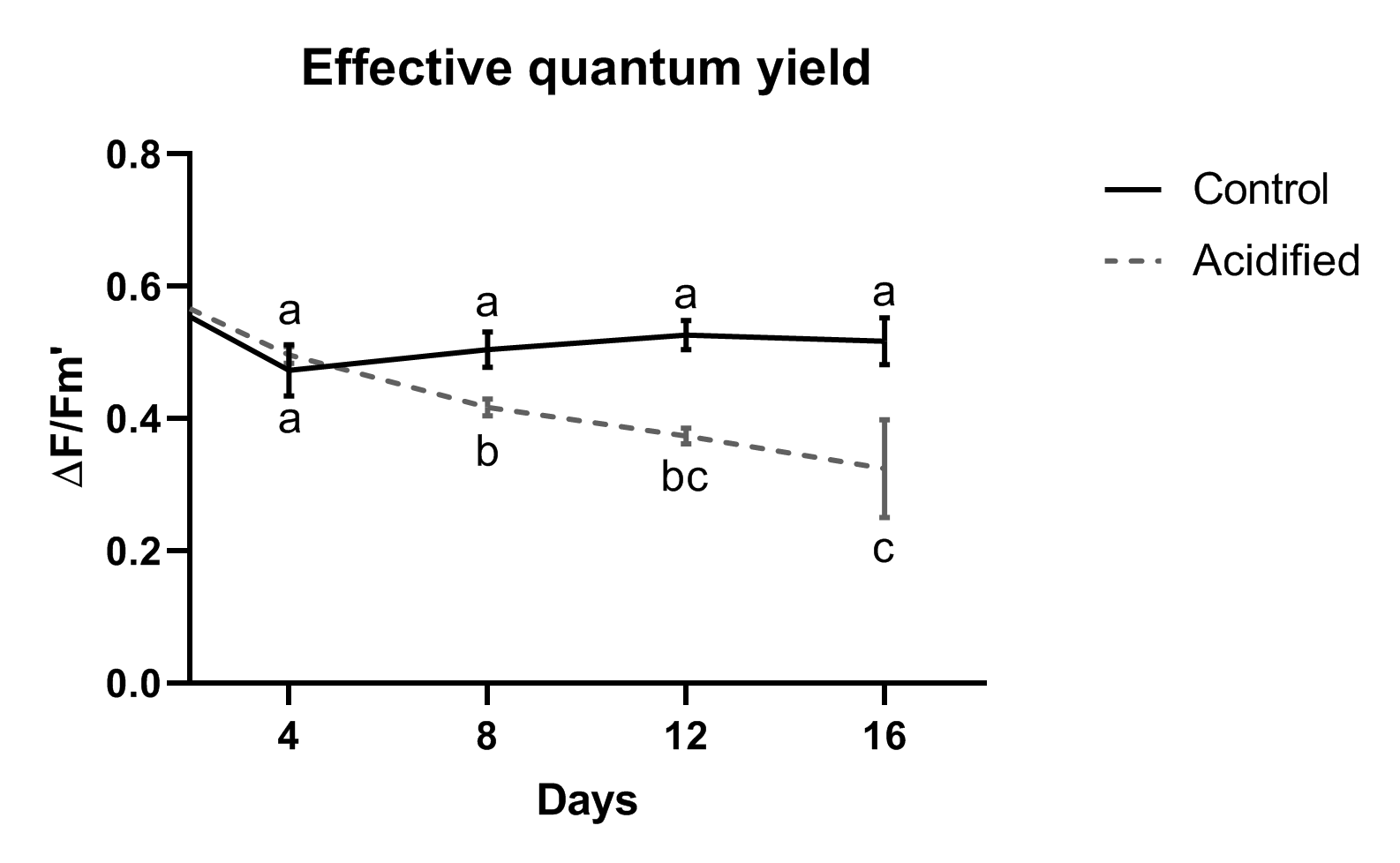

Supplement: S1 Fig — Measurements were made on control and acidified samples after 4, 8, 12 and 16 days of incubation. Effective quantum yield starts dropping henceforth 8 days of incubation and continues to diminish until the T4 (16 days), comparing to the control conditions (n = 6; p<0.001). (TIF) [file pone.0220130.s001.tif]

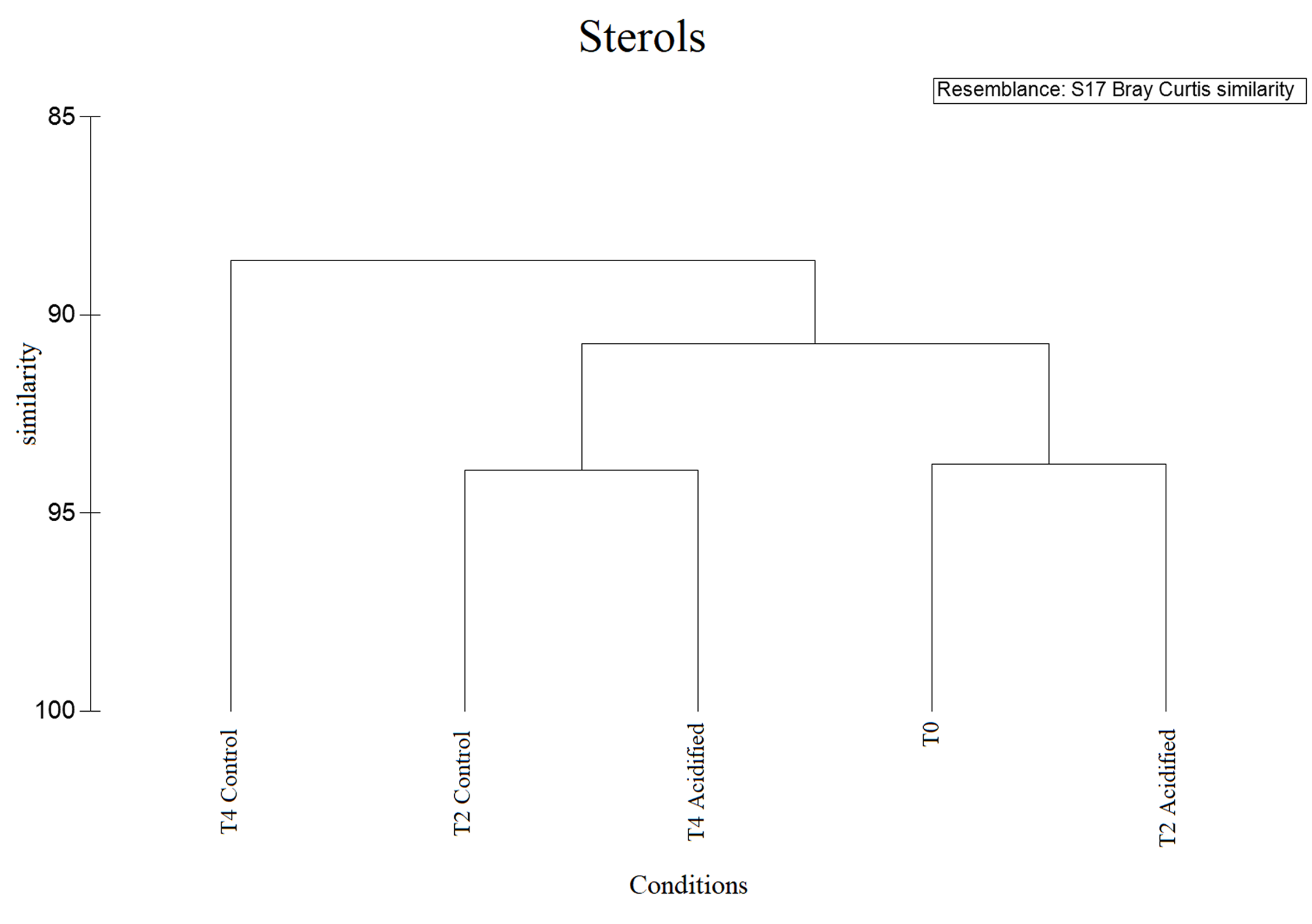

Supplement: S2 Fig — T0 = before assay samples, T2 = 8 days after incubation, T4 = 16 days after incubation. There was no similarity pattern amongst the samples. (TIF) [file pone.0220130.s002.tif]

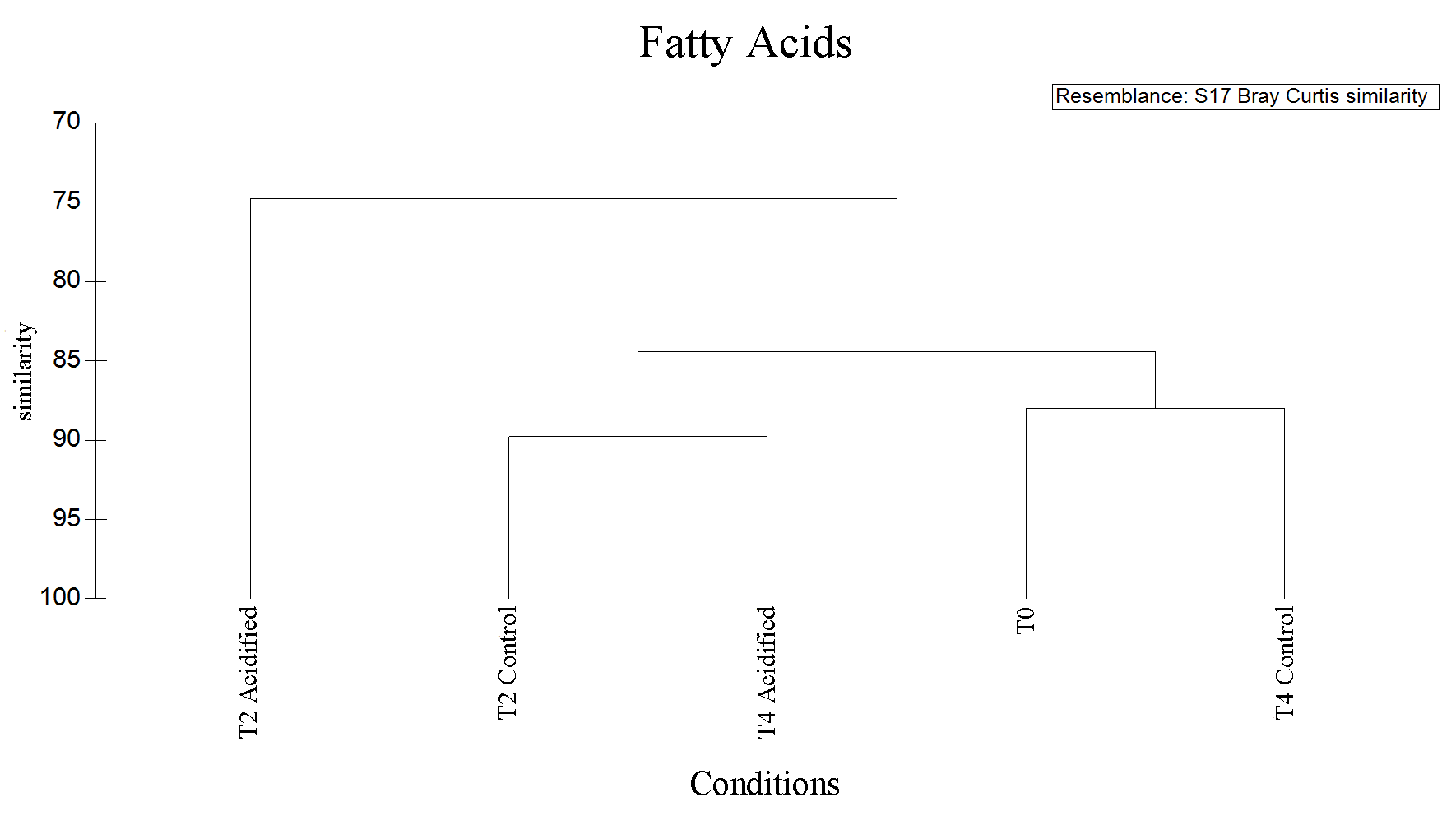

Supplement: S3 Fig — T0 = before assay samples, T2 = 8 days after incubation, T4 = 16 days after incubation. (TIF) [file pone.0220130.s003.tif]
