# Supplementary material for: Acidification-induced cellular changes in Symbiodinium isolated from Mussismilia braziliensis
Source: PLoS One. 2019 Aug 5;14(8):e0220130. doi: 10.1371/journal.pone.0220130 (PMC6681953; doi:10.1371/journal.pone.0220130)
Supplement: S1 Table — Statistical results of PERMANOVA. (DOCX) [file pone.0220130.s004.docx]

**S1 Table. Photosynthetic yeld.** Statistical results of PERMANOVA.

| Source of variation | gl | MS | Pseudo-F | P-value |
| --- | --- | --- | --- | --- |
| pH | 1 | 1824.7 | 73.556 | <0.001 |
| Time | 3 | 116.9 | 4.7137 | 0.0036 |
| pH x Time | 3 | 253.47 | 10.218 | <0.001 |
| Residual |  | 24.806 |  |  |
|  |  |  |  |  |
|  |  |  |  |  |
