# Supplementary material for: Acidification-induced cellular changes in Symbiodinium isolated from Mussismilia braziliensis
Source: PLoS One. 2019 Aug 5;14(8):e0220130. doi: 10.1371/journal.pone.0220130 (PMC6681953; doi:10.1371/journal.pone.0220130)
Supplement: S2 Table — Statistical results of One-way Anova. (DOCX) [file pone.0220130.s005.docx]

**S2 Table. Cell density.** Statistical results of One-way Anova.

| ANOVA table | SS | DF | MS | F (DFn, DFd) | P value |
| --- | --- | --- | --- | --- | --- |
| Treatment (between columns) | 0.02138 | 2 | 0.01069 | F (2, 12) = 58.47 | P<0.0001 |
| Residual (within columns) | 0.002194 | 12 | 0.0001828 |  |  |
| Total | 0.02357 | 14 |  |  |  |
