# Supplementary material for: Acidification-induced cellular changes in Symbiodinium isolated from Mussismilia braziliensis
Source: PLoS One. 2019 Aug 5;14(8):e0220130. doi: 10.1371/journal.pone.0220130 (PMC6681953; doi:10.1371/journal.pone.0220130)
Supplement: S3 Table — A = before assay samples; B = control samples; C = acidified samples. (DOCX) [file pone.0220130.s006.docx]

**S3 Table. Statistical results of Tukey´s multiple comparisons post-hoc for cell density.** A = before assay samples; B = control samples; C = acidified samples.

| Tukey's multiple comparisons test | Mean Diff. | 95.00% CI of diff. | Significant? | Summary | Adjusted P Value |
| --- | --- | --- | --- | --- | --- |
| A vs. B | -0.07084 | -0.09365 to -0.04803 | Yes | **** | <0.0001 |
| A vs. C | 0.01605 | -0.006762 to 0.03887 | No | ns | 0.1875 |
| B vs. C | 0.08689 | 0.06408 to 0.1097 | Yes | **** | <0.0001 |
