# Supplementary material for: Acidification-induced cellular changes in Symbiodinium isolated from Mussismilia braziliensis
Source: PLoS One. 2019 Aug 5;14(8):e0220130. doi: 10.1371/journal.pone.0220130 (PMC6681953; doi:10.1371/journal.pone.0220130)
Supplement: S4 Table — Results of One-way Anova for saturation inside lipid droplets. (DOCX) [file pone.0220130.s007.docx]

**S4 Table – Lipid droplets saturation statistcs.** Results of One-way Anova for saturation inside lipid droplets.

| ANOVA table | SS | DF | MS | F (DFn, DFd) | P value |
| --- | --- | --- | --- | --- | --- |
| Treatment (between columns) | 127767 | 2 | 63883 | F (2, 44) = 245.2 | P<0.0001 |
| Residual (within columns) | 11463 | 44 | 260.5 |  |  |
| Total | 139229 | 46 |  |  |  |
