# Supplementary material for: Acidification-induced cellular changes in Symbiodinium isolated from Mussismilia braziliensis
Source: PLoS One. 2019 Aug 5;14(8):e0220130. doi: 10.1371/journal.pone.0220130 (PMC6681953; doi:10.1371/journal.pone.0220130)
Supplement: S5 Table — A = before assay samples; B = control samples; C = acidified samples. (DOCX) [file pone.0220130.s008.docx]

**S5 Table. Post hoc Tukey´s multiple comparisons results for saturation inside lipid droplets**. A = before assay samples; B = control samples; C = acidified samples.

| Tukey's multiple comparisons test | Mean Diff. | 95.00% CI of diff. | Significant? | Summary | Adjusted P Value |
| --- | --- | --- | --- | --- | --- |
| A vs. B | 12.04 | -2.947 to 27.02 | No | ns | 0.1373 |
| A vs. C | -99.37 | -114.4 to -84.39 | Yes | **** | <0.0001 |
| B vs. C | -111.4 | -124.5 to -98.36 | Yes | **** | <0.0001 |
